# Ictal neural oscillatory alterations precede sudden unexpected death in epilepsy

**DOI:** 10.1093/braincomms/fcac073

**Published:** 2022-03-25

**Authors:** Bin Gu, Noah G. Levine, Wenjing Xu, Rachel M. Lynch, Fernando Pardo-Manuel de Villena, Benjamin D. Philpot

**Affiliations:** 1 Department of Neuroscience, Ohio State University, Columbus, OH, USA; 2 Department of Cell Biology and Physiology, University of North Carolina, Chapel Hill, NC, USA; 3 Neuroscience Center, University of North Carolina, Chapel Hill, NC, USA; 4 Electrical and Computer Engineering Program, Ohio State University, Columbus, OH, USA; 5 Department of Physiology and Cell Biology, Ohio State University, Columbus, OH, USA; 6 Department of Genetics, University of North Carolina, Chapel Hill, NC, USA; 7 Lineberger Comprehensive Cancer Center, University of North Carolina, Chapel Hill, NC, USA; 8 Carolina Institute for Developmental Disabilities, University of North Carolina, Chapel Hill, NC, USA

**Keywords:** sudden unexpected death in epilepsy, Collaborative Cross mice, ictal state, brain oscillations, phase-amplitude coupling

## Abstract

Sudden unexpected death in epilepsy is the most catastrophic outcome of epilepsy. Each year there are as many as 1.65 cases of such death for every 1000 individuals with epilepsy. Currently, there are no methods to predict or prevent this tragic event, due in part to a poor understanding of the pathologic cascade that leads to death following seizures. We recently identified enhanced seizure-induced mortality in four inbred strains from the genetically diverse Collaborative Cross mouse population. These mouse models of sudden unexpected death in epilepsy provide a unique tool to systematically examine the physiological alterations during fatal seizures, which can be studied in a controlled environment and with consideration of genetic complexity. Here, we monitored the brain oscillations and heart functions before, during, and after non-fatal and fatal seizures using a flurothyl-induced seizure model in freely moving mice. Compared with mice that survived seizures, non-survivors exhibited significant suppression of brainstem neural oscillations that coincided with cortical epileptic activities and tachycardia during the ictal phase of a fatal seizure. Non-survivors also exhibited suppressed delta (0.5–4 Hz)/gamma (30–200 Hz) phase-amplitude coupling in cortex but not in brainstem. A connectivity analysis revealed elevated synchronization of cortex and brainstem oscillations in the delta band during fatal seizures compared with non-fatal seizures. The dynamic ictal oscillatory and connectivity features of fatal seizures provide insights into sudden unexpected death in epilepsy and may suggest biomarkers and eventual therapeutic targets.

## Teaser

Utilizing Collaborative Cross mouse models of sudden unexpected death in epilepsy, Gu et al. report electrophysiological alterations in EEG power, phase-amplitude coupling, and connectivity in cortex and brainstem that distinguish fatal from nonfatal seizure. This study reveals potential pathophysiology of sudden unexpected death in epilepsy and provides targets for intervention.

## Graphical Abstract

**Graphical abstract:**
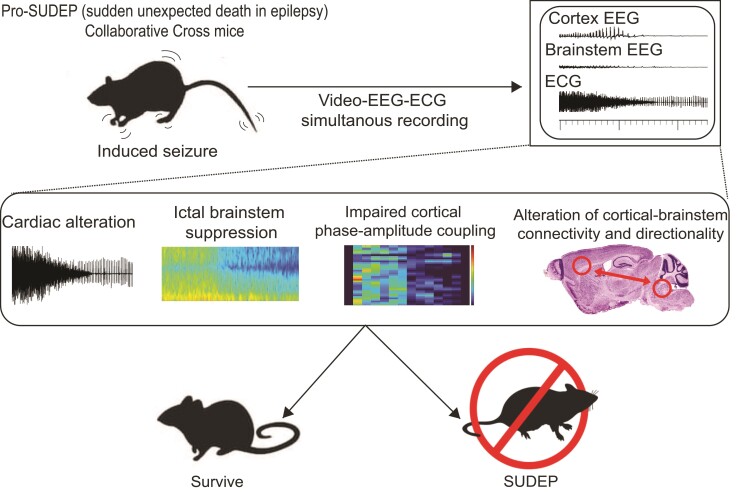

**See Gonzalez-Sulser (https://doi.org/10.1093/braincomms/fcac097) for a scientific commentary on this article.**

## Introduction

Sudden unexpected death in epilepsy (SUDEP) is a poorly understood fatal complication of epilepsy that poses a substantial public health burden and causes immeasurable familial loss.^1^ SUDEP is the most common cause of epilepsy-related mortality (∼1.3–1.65:1000/year)^2–4^ and is the second leading cause of death among neurological diseases after stroke when ranked by potential years of life lost.^5^ Despite the clinical importance and compelling need to understand SUDEP, the precise neurophysiological features that may distinguish fatal from non-fatal seizures remain elusive.^6^ Transformative prevention for SUDEP is needed and might be developed by understanding the neurologic events that accompany seizures leading to cardiorespiratory arrest. Because SUDEP has been rarely observed in epilepsy monitoring units, there is little informative data for studying its pathophysiology in humans, and almost nothing is known about the temporal and spatial neural pathophysiologic alterations that lead to SUDEP. Indeed, most previous efforts have focused on the postictal state,^7–10^ with little insight into potential oscillatory changes during the ictal state of fatal seizures. Finally, it is uncommon and challenging in the clinical setting to directly measure seizure-related neuronal activities from deep brain regions like the brainstem, a cardiorespiratory pace-making centre.

Animal studies remain essential for understanding the cause of epilepsy and SUDEP, as they offer a means to study a trait in a controlled environment. Animal models also provide a level of experimental testing and validation that is neither ethical nor possible in human subjects for the study of SUDEP. Utilizing the Collaborative Cross (CC), a mouse genetic reference population, which offers unprecedented genetic and phenotypic diversity,^11^ we serendipitously identified four CC strains that have enhanced mortality (>50%) immediately following a transient episode of generalized seizure induced by flurothyl.^12^ The identification of pro-SUDEP CC strains provides a unique opportunity for profiling the neural biomarkers and neurological events linked to SUDEP. Here, we monitored and analysed local field potentials (LFPs) recorded simultaneously from the primary motor cortex (M1) and dorsal raphe (DR) of brainstem together with cardiac function in freely moving mice during non-fatal and fatal seizures. DR was targeted in this study because it is the largest of the serotonergic nuclei,^13^ which plays a fundamental role in regulating arousal, cardiovascular, and respiratory activities, and has been implicated in the pathophysiology of SUDEP.^14–17^ We found quantitative spatial and temporal electrophysiological alterations in LFP power, phase-amplitude coupling, and connectivity in the cortex and brainstem that distinguish fatal from non-fatal seizures. Together, this information sheds light on potential pathophysiologic triggers of SUDEP and provides targets for therapeutic intervention in humans.

## Materials and methods

### Mice

Adult (P60–P120) male mice were used in this study. CC003/Unc, CC008/GeniUnc, CC009/Unc, and CC029/Unc (referred herein as CC003, CC008, CC009, and CC029, respectively) mice were acquired from the Systems Genetics Core Facility at the University of North Carolina at Chapel Hill between 2016 and 2020.^18^ The classic laboratory inbred C57BL/6J (B6J) mice (#000664) were purchased from The Jackson Laboratory and used as non-fatal controls because they are resistant to seizure-induced sudden death in flurothyl challenge. All mice were raised on standard mouse chow and kept on a 12:12 light/dark cycle. All studies were compliant with Institutional Animal Care and Use Committee protocols.

### ECG and echocardiogram monitoring in mice

ECG was performed using an Indus rodent system on mice anaesthetized with isoflurane. We used three-point contact for obtaining electrical signals of the cardiac rhythm and for determining heart rate and heart rate variability. A representative heart pattern was selected and ∼1000–1200 sequential heartbeats were processed and averaged to calculate the time elapsed between two successive R-waves (RR intervals). The heart rate variability was assessed by calculating the standard deviation of normal-to-normal (SDNN) RR intervals, coefficient of variation (CV) of the RR interval, and the root mean square of the successive differences (RMSSD) in RR interval using the same data. QT interval was measured from the beginning of the Q wave until the T wave returned to the isoelectric baseline. We calculated a rate-corrected QT interval (QT_c_) using the formula: QT_c_ = QT/(RR/100)^½^. Echocardiogram was measured on a gently restrained conscious mouse using a Vevo 2100 ultrasound. Parasternal views were used to obtain M-mode images for measuring left ventricle wall thicknesses, internal diameters at end-systole and end-diastole, ejection fraction, and fractional shortening. A representative film of the ultrasound was selected, and six heartbeat cycles of each measurement were taken to obtain an average per mouse.

### Flurothyl-induced seizure

Each mouse was placed in a 2 L glass chamber and allowed to habituate for 1 min before the top of the chamber was closed. 10% flurothyl (bis-2,2,2-trifluoroethyl ether; Sigma-Aldrich) in 95% ethanol was then infused at a rate of 200 µL/min onto a disk of filter paper suspended at the top of the chamber. The lid of the chamber was immediately removed to rapidly dissipate the flurothyl vapour and expose the mouse to fresh air after the emergence of the generalized seizure. Generalized seizure is defined by continuous rhythmic activities (>5 s) with elevation of LFP amplitude (2× baseline) coinciding with behavioural manifestation of whole body clonus and loss of postural control. A seizure can rapidly progress into a tonic extension of the hindlimb, at which point mice can either recover from seizure or experience sudden cardiorespiratory failure and die. All mice experienced a single generalized seizure before recovery or death. The time of death was defined as the point of abrupt irreversible and terminal cessation of cerebral electrical activity, which corresponded to the time of cessation of all movements, including chest wall as an indication of termination of respiration.^19,20^ Consistent with the previous observation, after terminal cessation of the brain and respiratory activities, the heart continued beating with a lower rate and amplitude for ∼2–7 more minutes before asystole.^21–24^

### Surgery

Mice were anaesthetized using isoflurane. Custom stainless steel bipolar recording electrodes were implanted in the right M1 (coordinates from bregma: 1.0 mm AP, 1.5 mm ML, and 1.0 mm below dura) and DR of brainstem (through the calvarium, AP −4.4 mm, ML 0 mm, and 2.8 mm below dura). A ground electrode was fastened to a stainless steel screw positioned on the skull above the left olfactory bulb. Two ECG leads were sutured in place below the left diaphragm (positive lead) and on the xiphoid (negative lead) to approximate a Lead II arrangement. Electrodes positions were secured using dental cement.

### Video-LFP-ECG simultaneous recording

A tethered system with a commutator was used, allowing mice to roam freely within the test chamber and enabling time-locked video-LFP-ECG recording of mouse brain and heart functions in conjunction with behaviour. LFP recordings were amplified (1000×) using single-channel amplifiers, sampled at a rate of 1000 Hz, and filtered with 0.3 Hz high-pass and 60 Hz notch filters. ECG recordings were amplified (2000×) using single-channel amplifiers, sampled at a rate of 2000 Hz, and filtered with a low-pass filter setting of 200 Hz. All electrical data were digitized with a CED Micro1401 and collected using Spike2 software.

### Signal processing and analyses

The raw data were analysed using custom MATLAB algorithms and Brainstorm.^25^ Ictal phase was defined by the emergence of a generalized seizure to the cessation of body convulsions. LFP spectrograms were generated using a fast Fourier transform with bin sizes of 0.25 Hz. For LFP power spectrum, an artefact-free baseline period (∼100 s) before initiation of flurothyl infusion was manually identified and used for normalization of power. We used time-resolved phase-amplitude coupling with a sliding time window length of 4 s through Brainstorm. A comodulogram was generated in the ranges of frequency for phase (fP, 0.5–30 Hz)/frequency for amplitude (fA, 30–200 Hz), fP (0.5–4 Hz)/fA (30–70 Hz) of delta/low gamma, and fP (0.5–4 Hz)/fA (80–200 Hz) of delta/high gamma. The phase-locking value and Granger causality between M1 and DR were first computed against the spectrum of frequency (0.5–60 Hz, in 1 Hz bin). The average phase-locking value and Granger causality were then calculated in the delta (0.5–4 Hz), theta (5–8 Hz), alpha (9–12 Hz), beta (13–29 Hz), and gamma (30–60 Hz) bands. ECG was analysed using Brainstorm by detecting the R peak time stamp during baseline, ictal, and postictal (10 s after termination of generalized seizure of survivors or death of non-survivors) periods. Instant heart rate time series were analysed using MATLAB with moving average in 1 s bin.

### Statistical analyses

All experiments and analyses were performed blind to strains. The Shapiro-Wilk normality test was performed to justify any parametric statistical tests that assumed Gaussian distribution. If data failed the normality test, we applied the appropriate non-parametric statistical tests instead. Unless otherwise noted, comparisons were analysed using two-tailed, unpaired Student’s *t*-test, one-way or two-way ANOVA with Šídák’s or Tukey’s *post hoc* test. *P* < 0.05 was considered significant. For *in vivo* electrophysiology studies, no significant differences were found in parameters measured during non-fatal seizures recorded between CC008 (*n* = 4) and B6J (*n* = 3) mice, thereby the data were pooled in the survivors’ group as discussed in detail below.

### Data availability

All MATLAB algorithms used in this study are available in Github (https://github.com/noahgl123/SUDEP). Other study data are available from the corresponding author, upon reasonable request.

## Results

### Cardiac function of pro-SUDEP CC strains

**Figure 1:**
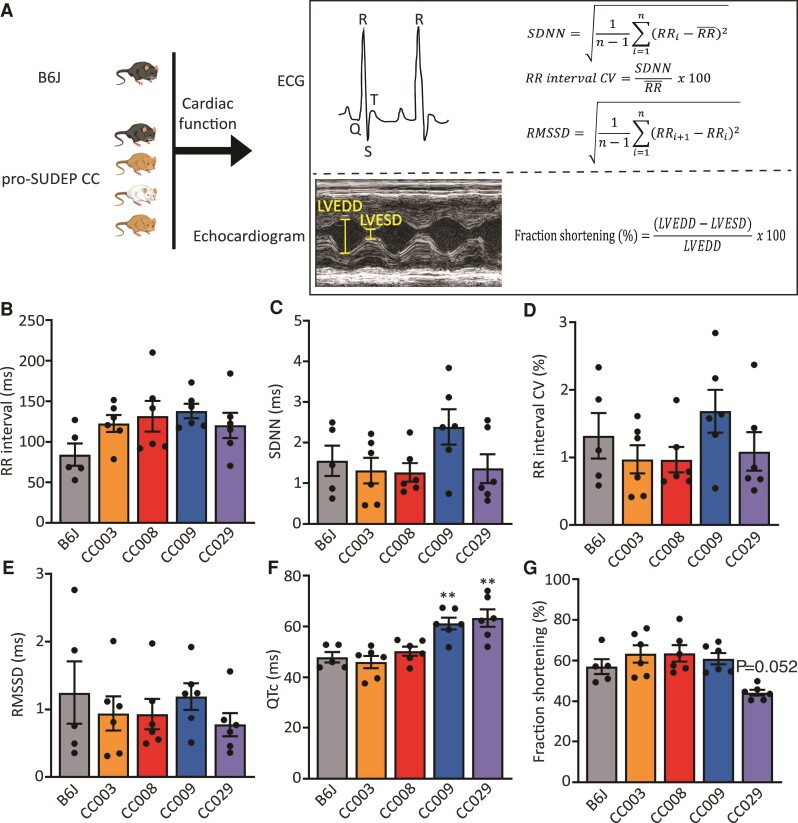
**Cardiac characterization of pro-SUDEP CC strains.** (**A**) Schematic of cardiac function measurements in B6J and pro-SUDEP CC mice using ECG and echocardiogram. (**B**) RR interval (*P* = 0.1258), (**C**) SDNN (*P* = 0.1500), (**D**) CV of RR interval (*P* = 0.3030), (**E**) RMSSD (*P* = 0.7106), (**F**) QTc, and (**G**) left ventricular fractional shortening in CC003, CC008, CC009, CC029, as well as B6J mice. Compared with B6J, CC009 (*P* = 0.0048) and CC029 (*P* = 0.0011) have prolonged QT interval. Data are presented as individual animal data points plus mean ± SEM. Data are analysed using one-way ANOVA with *post hoc* Tukey’s multiple comparisons test, *n* = 5–6, **P < 0.01. LVEDD, left ventricular end-diastolic diameter; LVESD, left ventricular end-systolic diameter.

Our previous screen identified four CC mouse strains (CC003, CC008, CC009, and CC029) that frequently (>50%) succumbed to sudden death immediately following an episode of transient generalized seizure induced by flurothyl,^12^ providing potential models to study seizure-induced sudden death on command in adult mice. Because SUDEP shares similar features with sudden cardiac death,^26,27^ we first characterized the baseline cardiac function in all four strains of pro-SUDEP CC mice as well as B6J mice as controls (Fig. 1A). ECG recordings of naive pro-SUDEP CC and B6J mice revealed similar heart rate (*P* = 0.1258) and characteristic heart rate intervals including the SDNN (*P* = 0.1500), CV of the RR interval (*P* = 0.3030), and RMSSD (*P* = 0.7106), suggesting invariant heart rate variability (Fig. 1B–E). However, CC009 and CC029 mice exhibited a significantly long QT interval compared with B6J mice (*P* = 0.0048 and *P* = 0.0011, respectively; Fig. 1F), which is a risk factor of cardiac arrhythmia and sudden death.^28–30^ Echocardiogram revealed that CC029 mice also have a trend towards decreased left ventricular fractional shortening (*P* = 0.0520; Fig. 1G), suggesting the myocardial contractility of CC029 may be impaired. Collectively, these results show that several CC mouse models exist for SUDEP, which likely arises through diverse mechanisms across these strains. In this study, we decided to prioritize CC008 of the SUDEP susceptible strain for the initial study as these mice did not exhibit overt cardiac abnormality, suggesting we could gain insights into the poorly understood non-cardiac contributions to SUDEP. CC009 and CC029 can be leveraged in the future to study the cardiac components of SUDEP.

### Suppressed ictal brainstem oscillations precede seizure-induced sudden death

**Figure 2:**
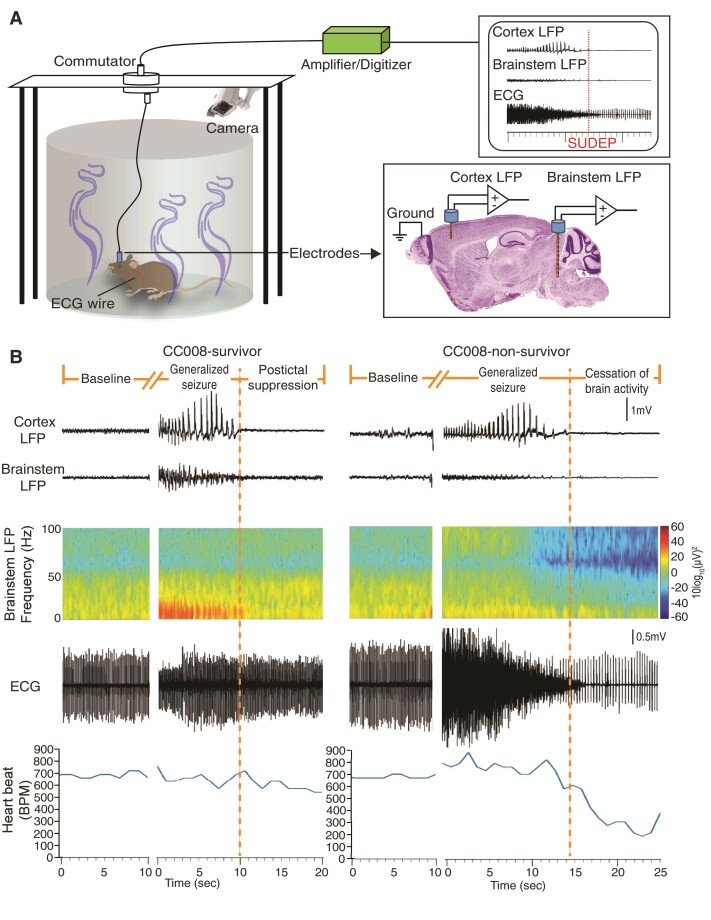
**Distinct brain and heart activities during ictal and postictal phases between non-fatal and fatal seizures.** (**A**) Schematic of LFP and ECG simultaneous recording in freely moving mouse during flurothyl challenge. (**B**) Representative LFP of cortex and brainstem (along with its spectrogram) as well as ECG recordings (along with instant heart rate time series) during baseline, ictal, and postictal states in CC008 survival and non-survival mice following flurothyl-induced generalized seizures.

**Figure 3:**
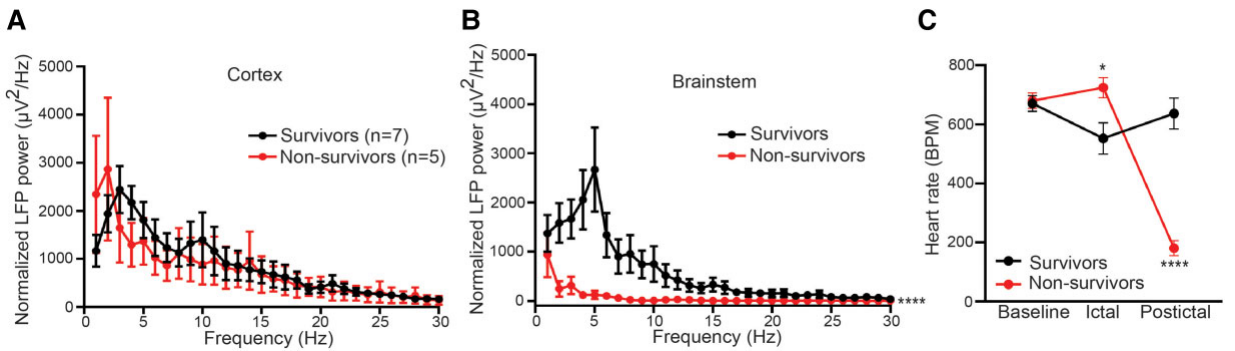
**Suppressed ictal brainstem oscillations precede SUDEP.** Normalized power spectrum of ictal cortex (**A**, *P* = 0.4615) and brainstem (**B**, *P* < 0.0001) of survivors (*n* = 7) and non-survivors (*n* = 5). (**C**) Heart rate during baseline (*P* = 0.9982), ictal (*P* = 0.0212), and postictal (*P* < 0.0001) phases of survivors (*n* = 7) and non-survivors (*n* = 5). Data are presented as mean ± SEM and analysed using two-way ANOVA with Šídák’s *post hoc* test. *P < 0.05; ****P < 0.0001.

The structural and functional alterations of brainstem play a fundamental role in regulating peri-ictal and ictal pathophysiological cascades that lead to SUDEP.^31–35^ However, the understanding of the temporal and spatial neuronal oscillations and connectivities between the forebrain and hindbrain in fatal seizures is still rudimentary. To examine the dynamic biopotential changes simultaneously in both M1 of cortex and DR of brainstem along with behavioural and cardiac function during fatal or non-fatal seizures, we monitored freely moving CC008 as well as B6J mice using a custom time-locked video-LFP-ECG recording system (Fig. 2A). We found electrographic seizures prevailed in both the cortex and brainstem during non-fatal seizures. Whereas strong ictal brainstem suppression coincided with cortical electrographic seizures preceding the cessation of brain activities and cardiorespiratory failure (Fig. 2B). Spectrogram and average baseline-normalized power spectrum revealed a significant ictal brainstem (*P* < 0.0001) but not cortical (*P* = 0.4615) suppression during fatal seizures compared with non-fatal seizures regardless of strain background (Fig. 2B, Fig. 3A and B). The suppression of ictal brainstem LFP power was generalizable across wavebands (1–60 Hz) with the most significant reduction in the beta oscillations (*P* = 0.0074) (Supplemental Fig. 1). In parallel to brain activity analysis, concurrent ECG monitoring suggests the non-survivors also experienced elevated (*P* = 0.0212) heart rate (725 ± 34 b.p.m.) during the ictal phase compared with survivors (553 ± 53 b.p.m.). Consistent with previous findings,^21–24^ the heart rate of survivors remained at baseline level during the postictal period, while that of non-survivors dropped drastically (181 ± 25 b.p.m., *P* < 0.0001) and continued after cessation of breathing and brain activity for an additional 2–7 min before asystole (Figs 2B and3C).

### Ictal cortical phase-amplitude coupling reduction during fatal seizures

**Figure 4:**
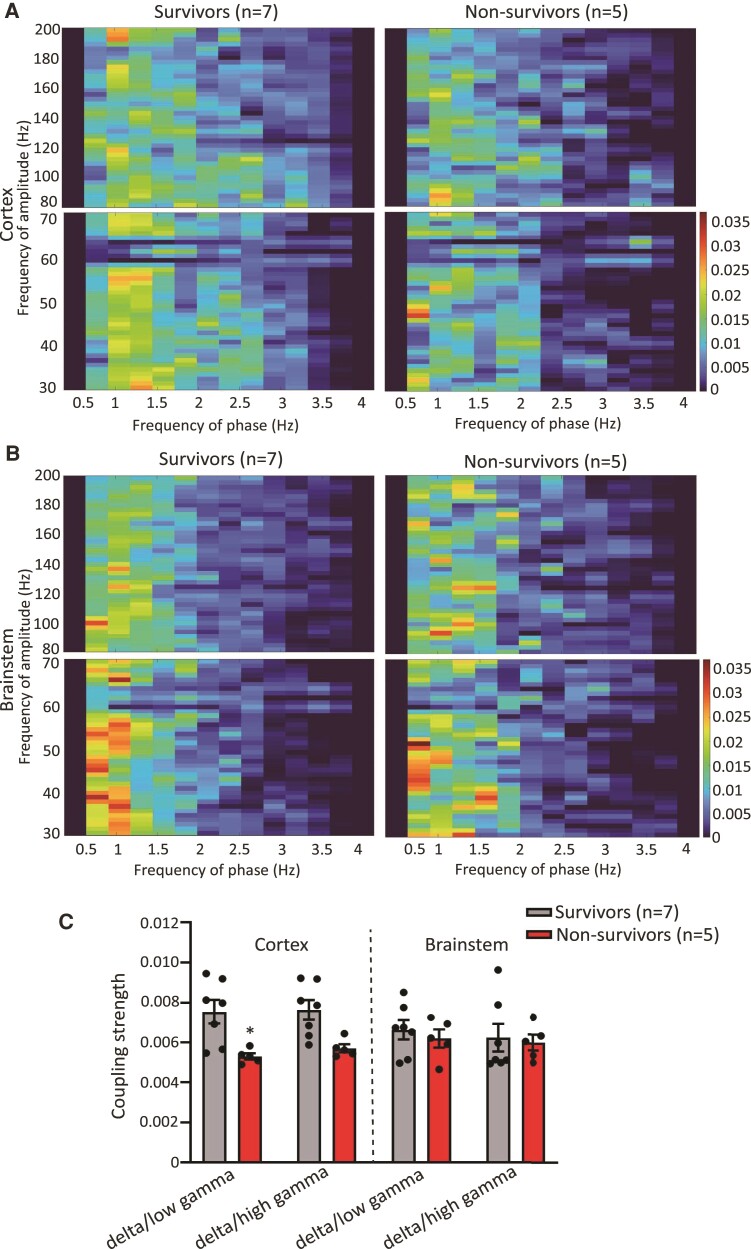
**Ictal cortical time-resolved phase-amplitude coupling reduction during fatal seizures.** (**A**) and (**B**) Average comodulogram of ictal cortical (**A**) and brainstem (**B**) phase-amplitude coupling in the delta/low gamma (fP: 0.5–4 Hz/fA: 30–70 Hz, bottom row) and delta/high gamma (fP: 0.5–4 Hz/fA: 80–200 Hz, top row) ranges of survivors (*n* = 7) and non-survivors (*n* = 5). (**C**) Average ictal cortical and brainstem phase-amplitude coupling strength within the delta/low gamma (cortex, *P* = 0.0174; brainstem, *P* = 0.9628) and delta/high gamma (cortex, *P* = 0.0513; brainstem, *P* = 0.9962) ranges of survivors (*n* = 7) and non-survivors (*n* = 5). Data are presented as individual animal data points plus mean ± SEM. Data are analysed using two-way ANOVA with Šídák’s *post hoc* test; *P < 0.05.

The synchrony between specific neuronal rhythms can provide insights into brain function and can be assessed using cross-frequency coupling. In particular, the coupling of the delta (0.5–4 Hz) and gamma oscillations (>30 Hz) was previously successful in localizing the epileptogenic zone of patients and in differentiating various states and types of seizures.^36–38^ One SUDEP case study suggested the disappearance of ictal phase-amplitude coupling dynamics recorded from the frontal temporal lobe, a feature that is normally present in epileptic survivors.^39^ We found across the broadband (fP: 0.5–30 Hz, fA: 30–200 Hz) phase-amplitude coupling, there is an overall strong delta (fP: 0.5–4 Hz):gamma (fA: 30–200 Hz) coupling in both cortex and brainstem during generalized seizures regardless of whether or not it is lethal (Supplemental Fig. 2). Thus, we decided to focus on analysing the strength of coupling between the delta phase against low-gamma (30–70 Hz) and high-gamma (80–200 Hz) amplitude, respectively. We found the strength of delta:low gamma and delta:high gamma coupling was reduced in cortex during fatal seizures compared with non-fatal seizures by 43.0% (*P* = 0.0174) and 32.3% (*P* = 0.0513), respectively (Fig. 4A and C). This impairment of coupling was not observed in the brainstem (delta:low gamma, *P* = 0.9628; delta:high gamma, *P* = 0.9962) (Fig. 4B and C).

### Elevated synchronization of cortical–brainstem network at delta band precedes seizure-induced sudden death

**Figure 5:**
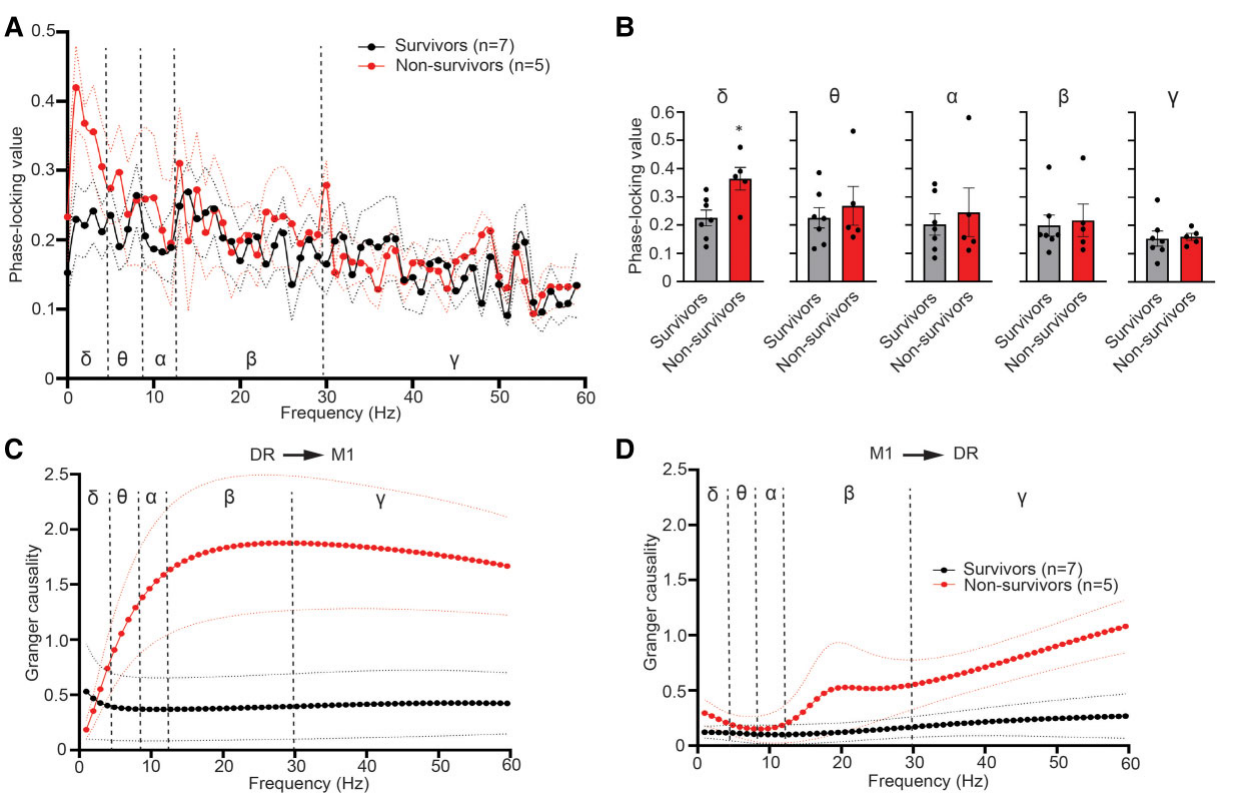
**Elevated synchronization of cortical-brainstem network at delta band precedes SUDEP.** (**A**) Phase-locking value spectrum and (**B**) averaged phase-locking value between ictal LFP from M1 and DR of survivors (*n* = 7) and non-survivors (*n* = 5) in the delta (δ, 0.5–4 Hz, *P* = 0.0148, Student’s *t*-test), theta (θ, 5–8 Hz, *P* = 0.8763, Mann–Whitney test), alpha (α, 9–12 Hz, *P* > 0.9999, Mann–Whitney test), beta (β, 13–29 Hz, *P* = 0.8763, Mann–Whitney test), and gamma (γ, 30–60 Hz, *P* = 0.8577, Student’s *t*-test) bands. *P < 0.05. Granger causality of ictal LFP (**C**) from DR to M1 (*P* < 0.0001, two-way ANOVA, survival factor) and (**D**) from M1 to DR (*P* < 0.0001, two-way ANOVA, survival factor), respectively, of survivors (*n* = 7) and non-survivors (*n* = 5). Data are presented as mean ± SEM (**A**, **C**, and **D**) or individual animal data points plus mean ± SEM (**B**). Data are analysed using two-way ANOVA (**A**, **C**, and **D**) and two-tailed unpaired Student’s *t*-test or Mann–Whitney test (**B**).

Abnormal connectivity of brain networks plays a fundamental role in epilepsy.^40,41^ We next examined the synchronization between M1 of cortex and DR of brainstem and explored frequencies at which synchrony occurs during the baseline and ictal states of fatal and non-fatal seizures. We found similar connectivity of M1 and DR during baseline recordings between survivors and non-survivors (Supplemental Fig. 3A and B). However, the phase-locking value significantly increased (*P* = 0.0148) in the delta range during fatal seizures compared with non-fatal seizures, suggesting increased delta phase synchrony between cortical–brainstem axis during seizures preceding sudden death (Fig. 5A and B). We further used Granger causality to determine the directional relationships between LFP recorded from M1 and DR in the context of seizure-induced sudden death. The directionality was indifferent during baseline recordings before non-fatal and fatal seizures (Supplemental Fig. 3C and D). Interestingly, compared with non-fatal seizures, ictal Granger causality spectra show a significant increase in the DR-M1 direction at alpha (2.8-fold increase, *P* = 0.0303), beta (3.8-fold increase, *P* = 0.0177), and gamma (3.3-fold increase, *P* = 0.0177) ranges and in the M1-DR direction at gamma (2.6-fold increase, *P* = 0.0480) band during fatal seizures. This bidirectional flow of information during fatal seizures also has influence from DR to M1 greater than the influence from M1 to DR (Fig. 5C and D and Supplemental Fig. 4).

## Discussion

SUDEP is clinically heterogeneous and has complex aetiology and genetic architecture. Single-gene manipulations of developmental epileptic encephalopathies (e.g., *SCN1A*, *SCN1B*, *SCN8A*, and *DEPDC5*) and long-QT syndromes (e.g., *KCNA1* and *KCNQ1*) have been used to model SUDEP.^6,42^ In these studies, the contribution of a single gene to SUDEP can be demonstrated, but these models lack consideration of the genetic complexity of the SUDEP response.^43–46^ We identified several novel inbred mouse strains with extreme sensitivity to seizure-induced sudden death, modelling SUDEP, and with likely different causal mechanisms. Compared with the monogenic mouse models of SUDEP, the large genetic diversity of CC better mimics the complex genetic causes of SUDEP in humans.^47^ In addition to canonical SUDEP-sensitive DBA/1J and DBA/2J inbred mouse strains, these pro-SUDEP CC strains provide basic scientists with valuable tools for mechanistic studies and the development and deployment of targeted therapeutics for prediction and prevention of SUDEP.

Despite the compelling need to understand the seemingly enigmatic nature of SUDEP, studies to identify the pathophysiologic underpinnings of this fatal complication of seizures are limited. The rare and unpredictable nature of SUDEP, along with the challenge of assessing electrical activity in deep brain regions, make it extremely challenging to discover the pathophysiologic triggers of SUDEP in humans. Retrospective reviews have identified some risk factors and pathologic alterations, but these studies suffer from a low number of cases and unclear causes of death.^48,49^ By combining a simultaneous video-LFP-ECG recording system in freely moving pro-SUDEP mice with power spectrum, synchrony, and connectivity analyses, we identified brain electrophysiological signatures that can be quantitatively measured as potential markers of SUDEP. To our knowledge, this is the first demonstration of the dynamic relationships between time series recorded from M1 and DR during baseline and ictal states in an animal model of SUDEP. The drop of brainstem power across frequencies during ictal state pushes the potential predictor of SUDEP earlier, before postictal generalized EEG suppression, the canonical marker of SUDEP, thereby providing an earlier intervention opportunity for SUDEP prevention. We postulate that a better understanding of ictal brain oscillation dynamics will suggest targets for therapeutic interventions by studying causal relationships in animal models using closed-loop electric or on-demand optogenetic rescue approaches.^50,51^

The finding of ictal brainstem suppression during fatal seizures is consistent with the critical role of serotonergic system of DR in regulating arousal, cardiovascular, and respiratory functions. Suppression of serotonergic tone was previously linked to fatal seizures.^52^ Indeed, pharmacological and optogenetic enhancement of DR serotonergic neurotransmission before seizure induction can reduce postictal generalized EEG suppression duration and block seizure-induced respiratory arrest.^15,16,53^ A previous study using single-unit recording of serotonergic neurons in DR of rats revealed variable firing with a mixture of increases and decreases during seizures induced by hippocampal electric stimulation, suggesting other types of neurons may also contribute to the overall suppression of DR population neuronal activity measured by LFP.^17^ Future work is needed to better understand the cellular contribution to SUDEP by using cell type-specific manipulations in DR. Direct current coupled recordings in anaesthetized transgenic mouse models of SUDEP also point to an active intermediary role of brainstem spreading depolarization in the cardiorespiratory collapse that leads to sudden mortality in epilepsy.^32–34^ Though the correlation between the direct current enabled infraslow recordings and the traditional alternating current enabled LFP and EEG recordings has not been clearly identified,^54,55^ application of inverse filter permits recovery of infraslow biosignals recorded using EEG^56^ so that both spreading depolarization and oscillation suppression can be incorporated into SUDEP assessment in humans. Integration of pulmonary function measurement is required for rigorous respiratory assessment to more fully reveal the relation and interaction of brain, heart, and respiration in the context of SUDEP.

The brain is a dynamical system with information exchange between distributed neuronal populations. Assessment of the extent of synchronization between these neuronal populations can shed light on the dynamic networks that underlie brain physiology and pathology. Synchronous oscillations in cortical circuits have been implicated in cognitive control, like attention and working memory.^57,58^ Neuronal synchrony measured by phase-locking value has also been utilized to predict seizures.^59,60^ Connectivity analysis suggests hypersynchrony of delta oscillation and elevated bidirectional information flow between M1 and DR during fatal seizures, which is consistent with the anatomical evidence and behavioural manifestation of tonic extension that precedes sudden death.^61–63^ This finding is also consistent with the observation that between-module connectivity is elevated in the brain of SUDEP patients using resting-state fMRI.^64^ This augmented connectivity may overload critical nuclei in the brainstem that may disrupt the integrity of brainstem networks.^65^ Though the precise functional roles of the ictal cortical-brainstem synchrony in mediating SUDEP sensitivity require further investigations, the brain connectivity readily measured using EEG, fMRI, and magnetoencephalography in humans makes brain synchrony a viable biomarker for SUDEP prediction.

## Supplementary Material

fcac073_Supplementary_Data

## Abbreviations

CV =: coefficient of variation
CC =: Collaborative Cross
DR =: dorsal raphe
LFPs =: local field potentials
M1 =: primary motor cortex
RMSSD =: root mean square of the successive differences
SDNN =: standard deviation of normal-to-normal
SUDEP =: sudden unexpected death in epilepsy
QT_c_ =: corrected QT interval
