# Correction to: Ictal neural oscillatory alterations precede sudden unexpected death in epilepsy

**DOI:** 10.1093/braincomms/fcac121

**Published:** 2022-05-13

This is a correction to: Bin Gu, Noah G. Levine, Wenjing Xu, Rachel M. Lynch, Fernando Pardo-Manuel de Villena, Benjamin D. Philpot, Ictal neural oscillatory alterations precede sudden unexpected death in epilepsy, *Brain Communications*, Volume 4, Issue 2, 2022, fcac073, https://doi.org/10.1093/braincomms/fcac073

In the originally published version of this manuscript, the name of the last author was incorrect. The last author's name should read “Benjamin D. Philpot” instead of “Benjamin D. Philpoti”

This error has been corrected. The publisher apologizes for the error.

