# Supplementary material for: Ictal neural oscillatory alterations precede sudden unexpected death in epilepsy
Source: Brain Commun. 2022 Mar 25;4(2):fcac073. doi: 10.1093/braincomms/fcac073 (PMC9035525; doi:10.1093/braincomms/fcac073)
Supplement: fcac073_Supplementary_Data [file fcac073_supplementary_data.pdf]

Supplemental materials for

Ictal neural oscillatory alterations precede sudden unexpected death in epilepsy

Bin Gu[1, 2, 3], Noah G. Levine[4], Wenjing Xu[2,5], Rachel M. Lynch[6],

Fernando Pardo-Manuel de Villena[6,7], and Benjamin D. Philpot[2,3,8]

[1]Department of Neuroscience, Ohio State University, Columbus, USA

[2]Department of Cell Biology and Physiology, University of North Carolina, Chapel Hill, USA

[3]Neuroscience Center, University of North Carolina, Chapel Hill, USA

[4]Electrical and Computer Engineering Program, Ohio State University, Columbus, USA

[5]Department of Physiology and Cell Biology, Ohio State University, Columbus, USA

[6]Department of Genetics, University of North Carolina, Chapel Hill, USA

[7]Lineberger Comprehensive Cancer Center, University of North Carolina, Chapel Hill, USA

[8]Carolina Institute for Developmental Disabilities, University of North Carolina, Chapel Hill, USA

Correspondence:

Bin Gu, Ph.D.

460 W 12th Avenue

612 Biomedical Research Tower

Columbus, OH 43210, USA

bin.gu@osumc.edu

This PDF file includes Supplemental Figures 1 to 4

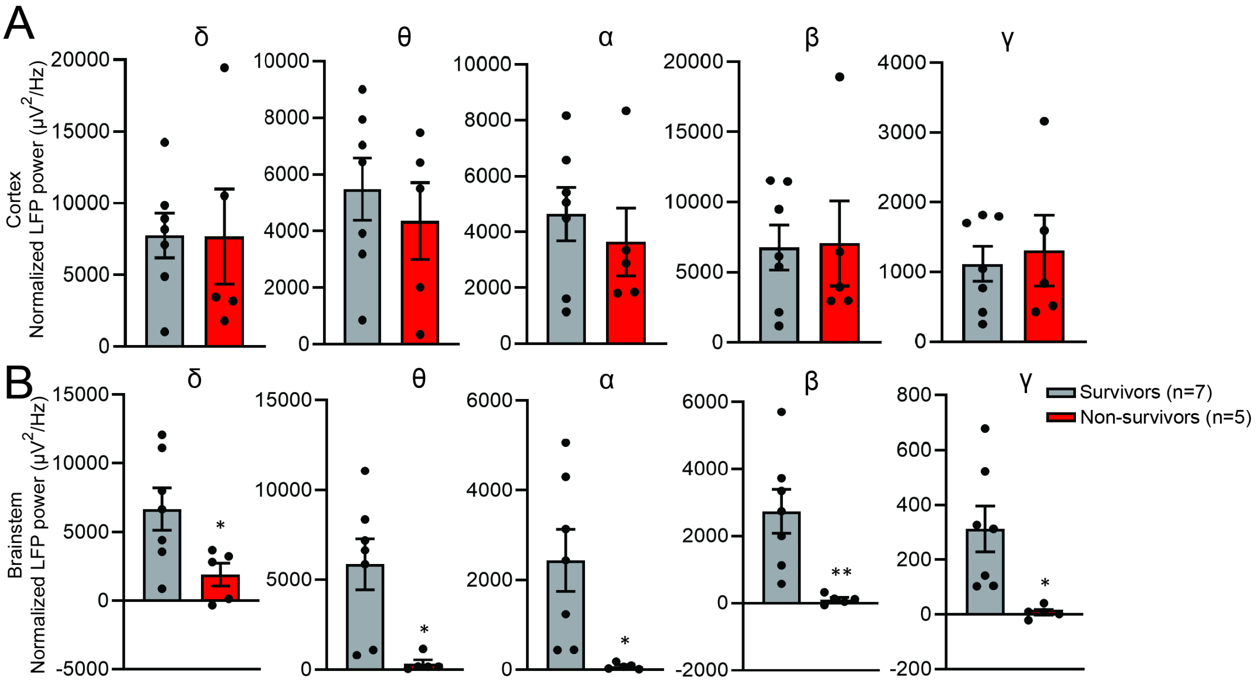

Supplemental Figure 1. (A) Normalized (subtracting baseline activities) ictal total LFP power in cortex of survivors (n = 7) and non-survivors (n = 5) in the delta (δ, 1–4 Hz, $P = 0.9840$, Student's t-test), theta (θ, 5–8 Hz, $P = 0.5290$, Student's t-test), alpha (α, 9–12 Hz, $P = 0.5298$, Student's t-test), beta (β, 13–29 Hz, $P = 0.9293$, Student's t-test), and gamma (γ, 30–60 Hz, $P = 0.7178$, Student's t-test) bands. (B) Normalized (subtracting baseline activities) ictal total LFP power in brainstem of survivors (n = 7) and non-survivors (n = 5) in the delta (δ, 1–4 Hz, $P = 0.0359$, Student's t-test), theta (θ, 5–8 Hz, $P = 0.0101$, Mann-Whitney test), alpha (α, 9–12 Hz, $P = 0.0178$, Student's t-test), beta (β, 13–29 Hz, $P = 0.0074$, Student's t-test), and gamma (γ, 30–60 Hz, $P = 0.0128$, Student's t-test) bands. Data are presented as individual animal data points plus mean ± SEM. Data are analyzed using two-tailed unpaired Student's t-test or Mann-Whitney test, * $P < 0.05$, ** $P < 0.01$.

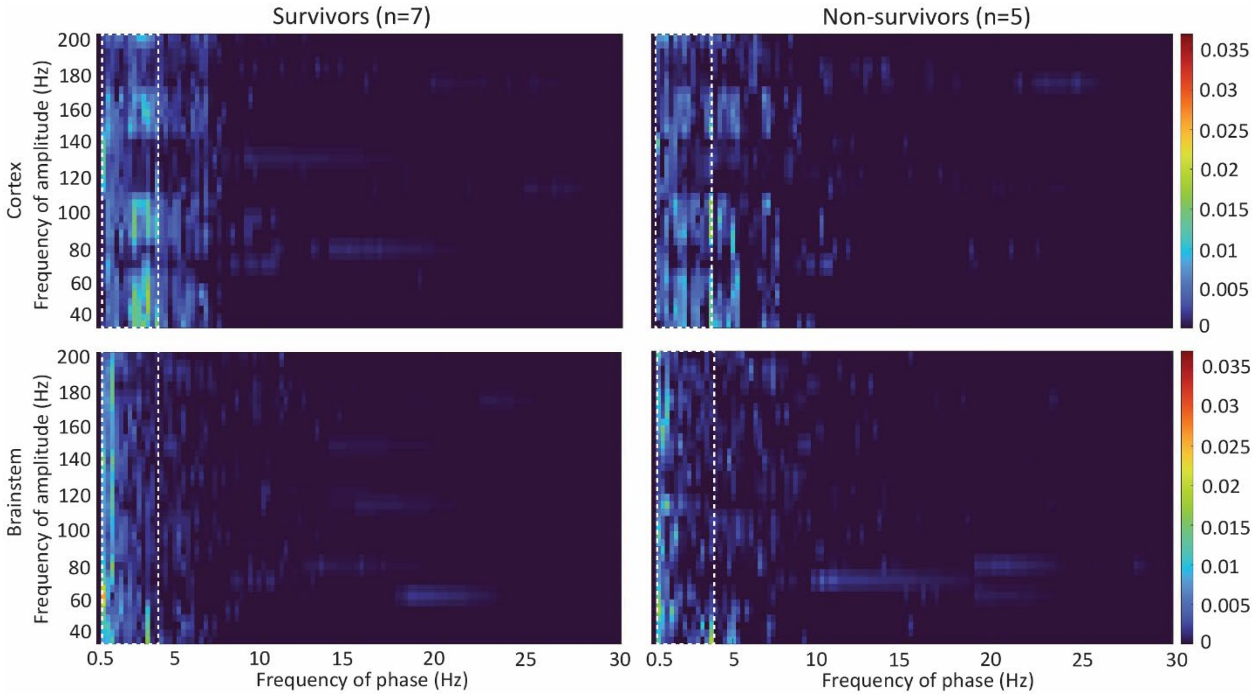

Supplemental Figure 2. Average comodulogram of ictal cortical (top row) and brainstem (bottom row) phase-amplitude coupling in the broader range (fP:0.5–30 Hz/fA:30–200 Hz) from survivors (n = 7, left column) and non-survivors (n = 5, right column). Dashed squares denote delta/gamma (fP:0.5–4 Hz/fA:30–200 Hz) coupling.

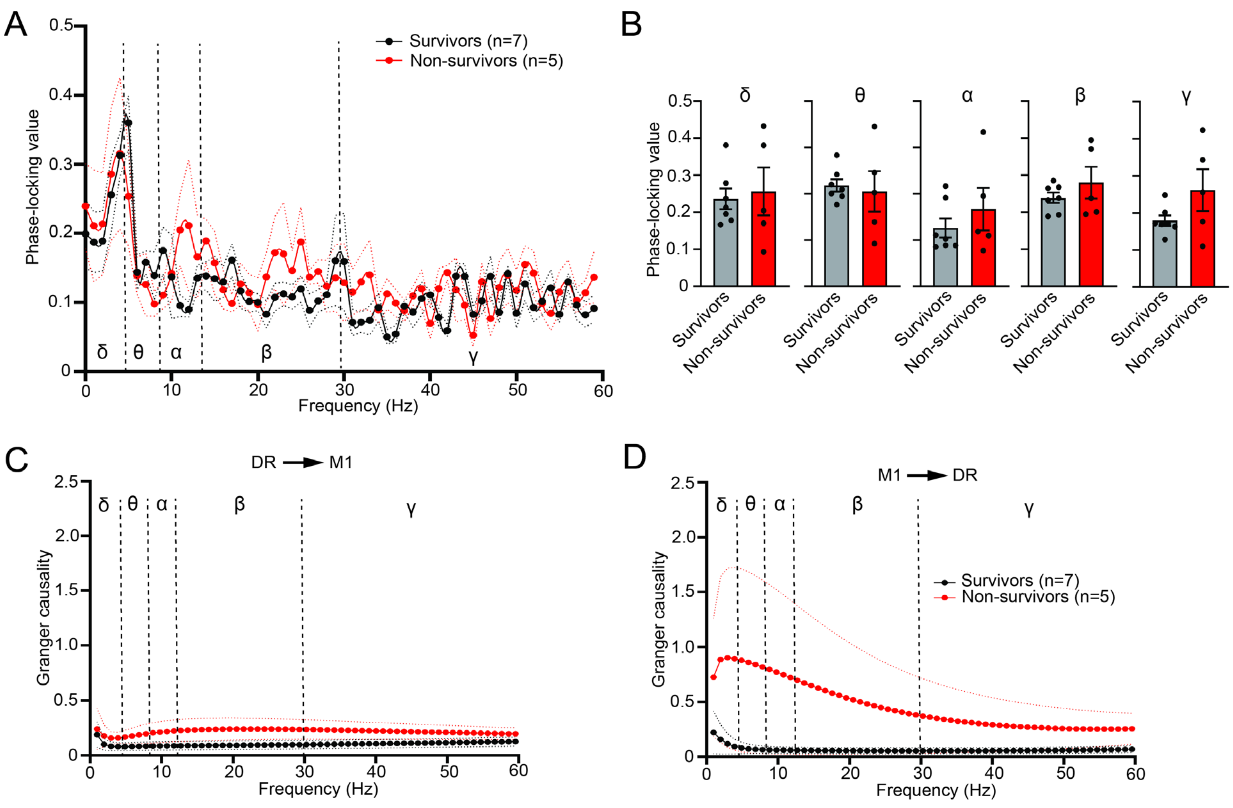

Supplemental Figure 3. (A) phase-locking value spectrum and (B) averaged phase-locking value between baseline LFP recorded from M1 and DR of survivors (n = 7) and non-survivors (n = 5) in the delta (δ, 0.5–4 Hz, *P* = 0.7537, Student's t-test), theta (θ, 5–8 Hz, *P* = 0.7508, Student's t-test), alpha (α, 9–12 Hz, *P* = 0.5303, Mann-Whitney test), beta (β, 13–29 Hz, *P* = 0.3197, Student's t-test), and gamma (γ, 30–60 Hz, *P* = 0.1257, Student's t-test) bands. Granger causality of baseline LFP recorded from DR to M1 (C) and from M1 to DR (D), respectively, of survivors (n = 7) and non-survivors (n = 5). Data are presented as mean ± SEM (A, C and D) or individual animal data points plus mean ± SEM (B).

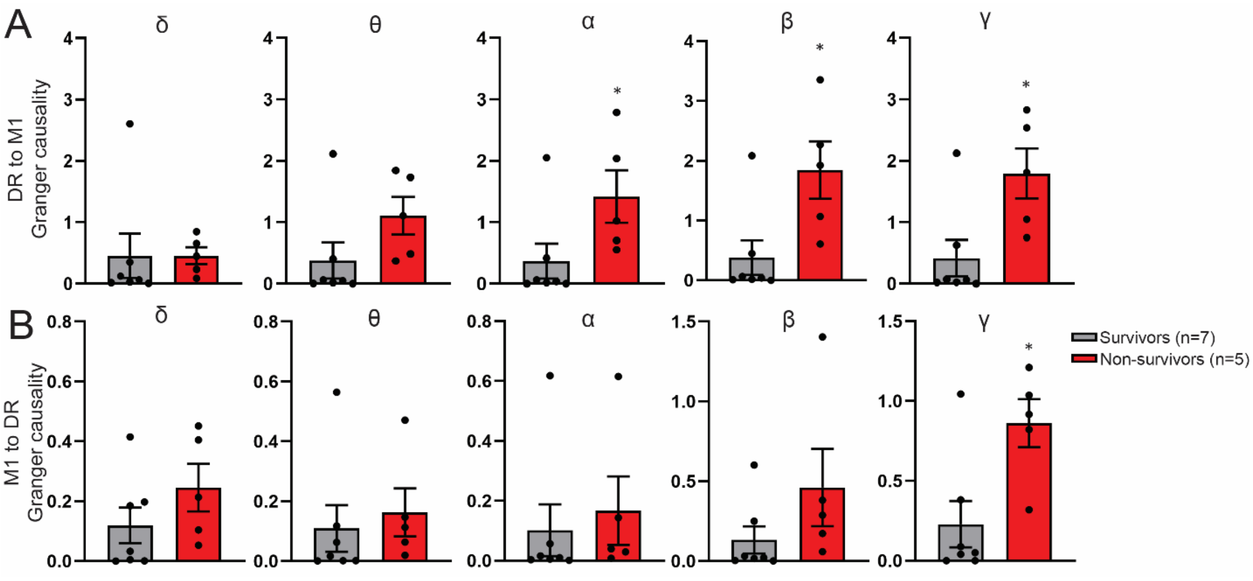

Supplemental Figure 4. (A) Granger causality of ictal LFP recorded from DR to M1 of survivors (n = 7) and non-survivors (n = 5) in the delta (δ, 0.5–4 Hz, $P = 0.1490$, Mann-Whitney test), theta (θ, 5–8 Hz, $P = 0.0732$, Mann-Whitney test), alpha (α, 9–12 Hz, $P = 0.0303$, Mann-Whitney test), beta (β, 13–29 Hz, $P = 0.0177$, Mann-Whitney test), and gamma (γ, 30–60 Hz, $P = 0.0177$, Mann-Whitney test) bands. (B) Granger causality of ictal LFP recorded from M1 to DR of survivors (n = 7) and non-survivors (n = 5) in the delta (δ, 0.5–4 Hz, $P = 0.1490$, Mann-Whitney test), theta (θ, 5–8 Hz, $P = 0.2020$, Mann-Whitney test), alpha (α, 9–12 Hz, $P = 0.2677$, Mann-Whitney test), beta (β, 13–29 Hz, $P = 0.0732$, Mann-Whitney test), and gamma (γ, 30–60 Hz, $P = 0.0480$, Mann-Whitney test) bands. Data are presented as individual animal data points plus mean ± SEM. Data are analyzed using Mann-Whitney test, * $P < 0.05$.
